# A Rare third ventricle solitary tuberculoma

**DOI:** 10.11604/pamj.2013.16.5.2815

**Published:** 2013-09-04

**Authors:** Hermann Adonis N'da, Adéréhime Haidara, Landry Drogba, Louis Kéhableon Derou, Serges Konan Yao, Vincent Ba Zézé

**Affiliations:** 1 Service de Neurochirurgie, Chu de Yopougon Abidjan, Côte D'Ivoire

**Keywords:** Intraventricular Tuberculoma, third ventricle, tuberculoma

## Abstract

The localization of the tuberculoma at the third ventricle is rare. The authors report a case of third ventricle solitary Tuberculoma which has occurred in a 10 year old patient and revealed by a syndrome of intracranial hypertension without tuberculosis stigma. This lesion appears clinically and radiologically as a primary brain tumor. A total removal using a subchoroidal approach to the third ventricle has been performed. Histological examination showed a tuberculous like granuloma. An adjuvant antituberculous chemotherapy practiced for 6 months brought the complete cure. The authors insist on the diagnostic and therapeutic difficulties in front of a third ventricle solitary tuberculoma.

## Introduction

Tuberculosis continues to be a public health problem in under developed world. Tuberculoma's rate in all intracranial space occupying lesions is variable according to countries and series [1], 15.9% for Arsenjo et al in Chile, 7.3% for Arseni et al in Romania; 12.5% for Odeku et al in Nigeria. The absence of clinical sign specific to the third ventricle tuberculoma makes the diagnosis difficult to establish apart from any tuberculous mark [2, 3]. Therapeutic management seems to be also difficult because of no veritable consensus on the treatment duration of this rare extra pulmonary form. This present report illustrates a rare case of tuberculoma which has mimed a primary intraventricular tumor and insist on diagnostic and therapeutic management difficulties.

## Patient and observation

A ten year old female has been allowed in hospitalization for intense frontal headaches. Theses headaches were associated to a progressive loss of the vision and to tonic seizure of the lowers members. These clinical signs had evolved for four (4) months. The neurological examination was normal. The examination of the other devices was also normal and there was no fever.

**Figure 1:**
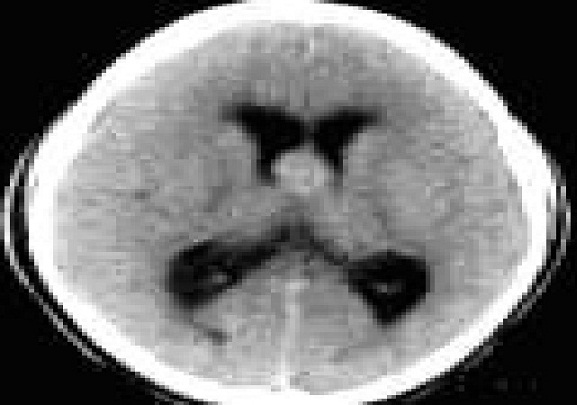
CT scan with an isodense nodular formation in the anterior part of third ventricle

**Figure 2:**
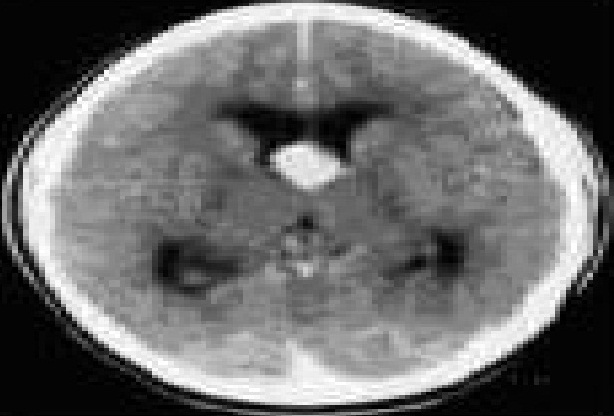
CT scan with contrast administration

Computed tomography scan (CT Scan) revealed a nodular formation (19 mm high on 16.7 mm wide) in the anterior part of the third ventricle with an obstructive hydrocephalus (Figure 1). This lesion was enhanced by the contrast administration (Figure 2). The likely diagnosis of intraventricular tumor (colloid cyst) was evoked. A total removal has been performed using a subchoroidal approach. The patient left the hospital 8 days after surgery.

**Figure 3:**
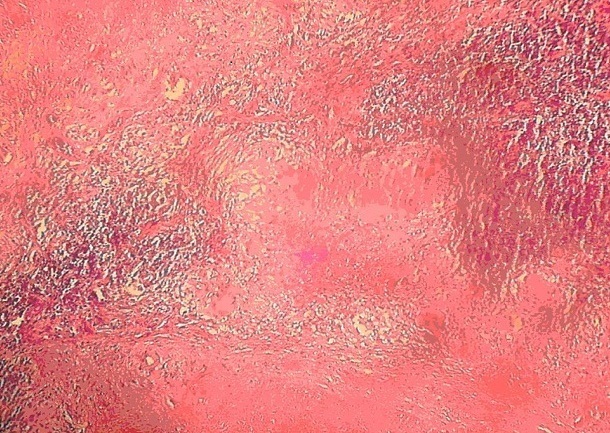
Haematoxylin Eosin (HE) × 100, Tuberculous granuloma with giants cells and caseous necrosis

Histological examination revealed the presence of central necrosis surrounded by a tuberculous like granuloma (Figure 3).

A new clinical status of the patient did not show tuberculosis infection stigma, HIV serology was negative. The Tuberculin skin test and the lung radiography were also normal. The confirmation of the tuberculous etiology was obtained by the Polymerase Chain Reaction (PCR). The CSF examination showed only 3 cells (3 lymphocytes) and the bacteriological examination using the ziehl-nielsen staining was negative. Protein level in CSF was normal (0.4g/l). She was treated with antituberculous chemotherapy including Isoniazid, Rifampicin, Pyrazinamid and Ethambutol for six months. The patient is living normally and is following her secondary studies, six (6) years afterwards.

## Discussion

The intraventricular localization of tuberculoma is rare. Primarily, tuberculoma seems to be child and young adults problem [4] because most of time, the patient do not exceed twenty years old.

The diagnostic strategy in front of a tuberculoma can be made of 2 manners. Firstly, the existence of a known or evolutionary bacillary context, having preceded or accompanying the neurological symptomatology, or the discovery of a tuberculous hearth, must firstly evoke the tuberculoma in front of an intracranial expansive process [5, 6].

This bacillary context did not exist in our case. The second case is the pure hypertensive form which appears clinically and radiologically as a brain primary tumor. The Diagnosis of this form is very difficult because of no bacillary context.

Even if neuroimaging can contribute to the diagnosis, it remains very difficult to differentiate the tuberculoma from a cerebral tumor. However, the peripheral catch of contrast, the central necrosis, a disproportionate oedema and the traction on the septum pellucidum are neuroimaging (CT Scan; MRI) features of intraventricular tuberculoma [1, 7].

The traction of the septum pellucidum is a very important radiological feature of tuberculoma because it signs the presence of intraventricular adhesive process [7, 8].

For many authors [4, 9–10], an antituberculous chemotherapy has to be establish without necessary biopsy when the diagnosis of tuberculoma is reasonably likely and outside quite intracranial pressure increase.

We have no doubt on efficacy of the antituberculous chemotherapy but in spite of a strong suspicion of tuberculoma, it could be confused with brain primary tumors. Furthermore, exclusive chemotherapy could be long (12 to 18 months) with 3 years extreme for Rossi et al [11]. This kind of duration exposes the patient to the toxicity of antituberculous drugs. We recommend surgical resection or when possible an endoscopy biopsy in front of accessible lesion to firstly diagnosis confirmation.

Complete resection allows reducing in six months the antituberculous treatment duration. According to CT scan evolution of the lesion, this period could be prolonged in 9 months. This is what's doing for extra pulmonary form (intracranial or vertebral location) of tuberculosis in our country, where, tuberculosis is an endemic disease.

## Conclusion

The third ventricle location of tuberculoma is rare. The diagnosis of the pure hypertensive form is very difficult and very often presumptive.

Surgery is recommended to confirm the diagnosis and to reduce chemotherapy duration and toxicity. For these reasons complete resection via open surgery or endoscopic surgery of this lesion when it is possible is desirable.
